# EXPLORING THE LINK BETWEEN SLEEP AND DEMENTIA: MULTIFACETED APPROACHES

**DOI:** 10.1093/geroni/igad104.0503

**Published:** 2023-12-21

**Authors:** Darina Petrovsky, Elizabeth Luth

## Abstract

Sleep and sleep quality have been linked to cognitive function over time. The quality of caregiver sleep can also affect their ability to function and provide support to persons living with dementia. This symposium leverages longitudinal survey data from two large US population-based surveys: the National Health and Aging Trends Study (NHATS) and the National Study of Caregiving (NSOC). This symposium aims to explore the relationship between sleep, cognitive function, and care activities for older adults living with and without dementia and their caregivers. First, Dr. Petrovsky will report findings that examined the relationship between caregiver sleep quality and persons living with dementia status using NHATS and NSOC data. Next, Dr. Esiaka will present cross-sectional findings from examining the relationship between older adult sleep quality, neighborhood disorder, and cognitive function using the NHATS data. Drs. Klingman and Sullivan will present the results of an analysis comparing the relationship between sleep and frequent hospitalization in persons living with dementia and those without dementia. Collectively, these papers will report on findings examining how a potentially modifiable factor, sleep, relates to cognitive function and caregiving.

